# An Integrated Fully Digital Prosthetic Workflow for the Immediate Full-Arch Restoration of Edentulous Patients—A Case Report

**DOI:** 10.3390/ijerph19074126

**Published:** 2022-03-31

**Authors:** Barbara Sobczak, Piotr Majewski

**Affiliations:** 1 Dr Sobczak Dental Clinic, 04-881 Warsaw, Poland; 2 Implant Dentistry Department, Dental Institute Collegium Medicum, Jagiellonian University, 31-155 Cracow, Poland; poczta@piotrmajewski.eu

**Keywords:** immediate placement, immediate loading, full-arch restoration, CAD CAM technology, digital implant dentistry

## Abstract

Digital prosthetic workflows may significantly increase the efficiency and predictability of the immediate rehabilitation of implant-supported fixed complete dentures. Advanced digital prosthetic workflows require exact and detailed virtual planning models. The direct generation of these models via direct digital impressions remains technique sensitive and demanding. This report illustrates an advanced digital workflow for accurate and efficient immediate full-arch restoration, with an aesthetically and anatomically adapted natural tooth-like prosthesis. The workflow application to fully edentulous arches, and arches with residual failing dentition, is presented. A key characteristic was efficiently integrating and interlinking the prosthetic and surgical workflows via denture replica surgical guides as landmarks for scan registration. This approach allowed for accurate implant placement and efficient and detailed anatomy-based chairside prosthetic planning, and for the manufacturing of the provisional and final restorations under detailed consideration of implant restoration, and the patient’s macro-aesthetic and soft tissue anatomy.

## 1. Introduction

Immediate, i.e., immediately placed and loaded implant-supported fixed complete dentures (IFCDs) represent a well-established and increasingly popular modality to treat partial or complete edentulism [1,2,3]. Digitally guided techniques have rendered the associated surgical protocols more predictable, efficient, and less invasive [4,5]. The digitalization of prosthetic planning and manufacturing, and the introduction of novel materials, have recently led to a substantial transformation of prosthetic workflows [6,7,8,9]. Advanced digital workflows increasingly allow for the creation of prosthetic frameworks with ideal smile aesthetics that are accurately adapted to patient-individual anatomic preconditions [10,11,12].

Advanced digital prosthetic workflows require exact and detailed virtual planning models that include detailed and accurate information on the implant positions, soft tissue anatomy, and occlusion [8,13]. The direct generation of virtual prosthetic models for restoring full arches, and specifically edentulous arches, has been described as challenging, and often requires a combination of multiple intraoral or extraoral scans. These scans span the entire arch and are needed to feed the model with accurate information on the occlusion and maxillomandibular relation. However, their registration is often impeded by the limited availability of anatomic landmarks [8,14,15,16]. Many approaches have been proposed to compensate for these limitations [17,18,19,20,21,22,23]. Examples include multi-staged or indirect workflows, while direct digital prosthetic workflow may require auxiliary scanning guides, fiducial markers, or pins as anatomic references to facilitate the scan registration. As such, these workflows remain complex, technique sensitive, and may limit clinical efficiency.

This case report presents an advanced and direct digital workflow for the immediate transition of conventional full dentures into a fixed full-arch restoration. The chairside prosthetic planning and manufacturing workflow presented here allows for the adaptation of the aesthetic coronal and soft tissue interfacing cervical aspects of the prosthetic framework to the patient’s aesthetics and local soft and hard-tissue anatomy. A special feature of the presented workflow is related to the efficient integration and interlink of the prosthetic and surgical workflows, which is based on specific denture replica surgical guides as landmarks for scan registration.

## 2. Materials and Methods

### 2.1. Diagnosis

**Figure 1:**
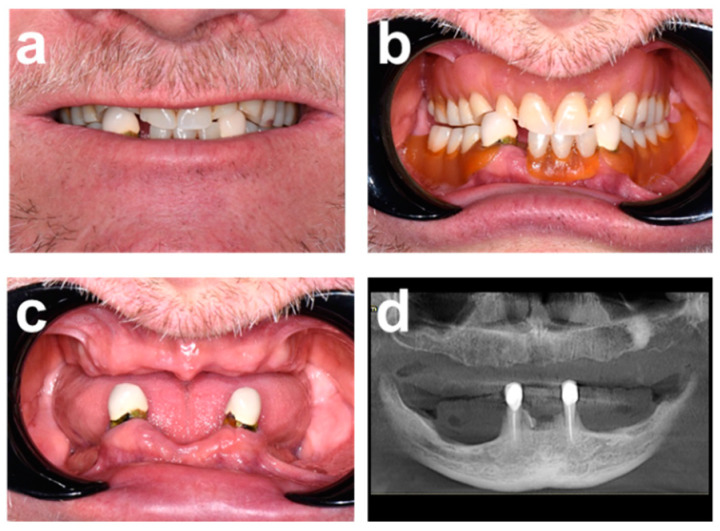
Initial clinical and radiographic examination. (**a**,**b**) Frontal views of the natural smile line and the conventional restoration using retractors. (**c**) Clinical presentation of the edentulous alveolar ridges and residual dentition. (**d**) Panoramic radiograph.

A 67-year-old male patient presented in our clinic (Sobczak Clinical Centre, Radosc, Poland) with a chief complaint related to the poor functionality and aesthetics of his conventional dentures. Initial comprehensive clinical (Figure 1a–c) and radiographic examination (Figure 1d) revealed a fully edentulous maxilla and a partially edentulous mandible. Residual canines #33 and #43 were overcrowned and endodontically treated, and presented with poor prognoses as part of planned restorative concepts (Figure 1c).

As illustrated by the frontal pictures and panoramic radiograph in Figure 1c,d, the patient’s maxillary alveolar crest was moderately atrophied, and it presented adequate vertical and horizontal bone volume for implant placement and a thick, healthy keratinized mucosa. The distal aspects of the mandibular alveolar crest were severely atrophied but presented osseous dimensions adequate for implant placement in the interforaminal zones.

The differential diagnostic evaluation indicated a lower lip line not revealing gingival margins, appropriate occlusal vertical dimensions, and the presence of a teeth-only defect allowing for reconstruction with a white bridge [12]. The patient’s general medical history was non-contributory to implant treatment. After discussing the benefits, risks, and alternative treatment options, the patient consented to rehabilitate both arches with immediate IFCDs using a direct, fully digital approach. Treatments were provided by a team of one oral surgeon and one digital dental technician.

### 2.2. Treatment Planning

The patient’s conventional dentures were relined, the correct fit was verified, and the dentures were modified with fiducial markers along the palatal and lateral rims (Gutta-Percha, DENTSPLY International Inc., York, PA, USA). Dual cone-beam computed tomography (CBCT) scans (HyperionX9, MyRay, Imola, Italy; Implant studio, 3Shape A/S, København, Denmark) of the patient with dentures and of the dentures alone were recorded. Individual scans were aligned using the software algorithm to obtain a three-dimensional model of the patient’s osseous anatomy and conventional dentures.

In the same session, both arches were scanned using an intraoral scanner (3Shape TRIOS^®^, 3Shape A/S, København, Denmark) to record the virtual occlusion, bite registration, and the occlusal vertical dimension (OVD). Further frontal photos of the patient smiling and wearing retractors were taken.

**Figure 2:**
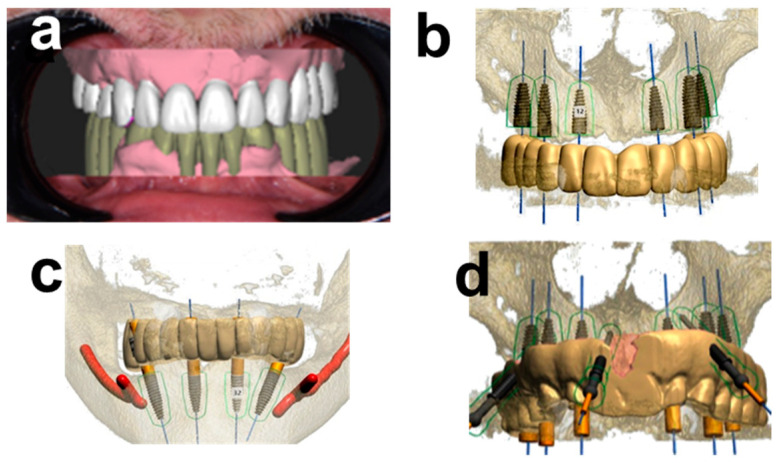
Prosthetic and surgical planning. (**a**) provisional wax-up of the maxillary and mandibular restorations after digital smile design (DSD). The provisional wax-up is shown in relation to the patient’s anatomy as represented by frontal retracted photographs. (**b**) Planned maxillary implant restoration and (**c**) mandibular implant restoration in relation to the provisional wax-up and (**d**) in relation to the denture-shaped anchoring pin guide (APG).

The standard tessellation language (STL) files from the intraoral scans (IOS) and frontal digital photographs were overlayed to derive diagnostic digital wax-ups that were adapted by digital smile design (DSD) (Figure 2a). Specifically, digital waxing and DSD considered the patient’s smile symmetry, i.e., the midline, curve of Spee, prosthetic plane, and existing gingival margin. The maxillo-mandibular relation of the patient wearing his conventional dentures were carefully evaluated by assessing the symmetric relation of facial thirds, as defined by the distances between the trichion and glabella, the glabella, and subnasale, and the subnasale and menton, respectively. The patient did not report any pain or discomfort related to his conventional restoration. Based on this assessment, a decision was taken to preserve the occlusal vertical dimension and correct only the occlusal plane of the future restoration. At this stage, any macro- and microaesthetic aspects of the smile, i.e., the smile line, teeth shape, length, and width, were redesigned using examples from the software library (in-CAD Smile Creator, exocad GmbH, Darmstadt, Germany). The occlusal plane was further corrected while preserving the vertical dimensions.

Digital imaging and communication in medicine (DICOM) data from CBCT were superimposed with standard tessellation language (STL) files from intraoral scans of the conventional denture as a reference and the diagnostic wax-up after DSD, respectively. From the resulting virtual model, it was possible to plan the ideal prosthetically guided position of the implants. Specifically, the as-derived first-molar-to-first-molar white bridges in the maxilla and mandible were planned on six axially-placed tapered implants in the maxilla, and two axially and two inclined distal implants in the mandible (Figure 2b,c). Recent evidence supports that the latter resulting cantilevered mandibular zirconia-based full-arch restoration with distally inclined implants represents a biomechanically sound treatment option with an adequate long-term survival prognosis [24,25]. Figure 2d illustrates the planned implant restoration relative to the anchoring pin guide (APG) (Implant studio, 3Shape A/S, Copenhagen, Denmark).

### 2.3. Surgical Procedure

**Figure 3:**
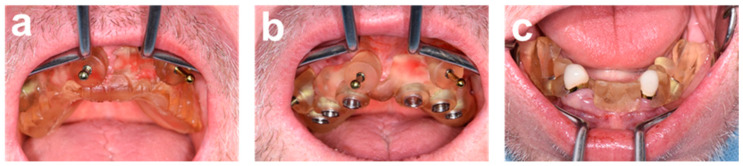
Illustration of the surgical templates for guided osteotomy preparation and implant placement. (**a**,**b**) Chairside printed maxillary guides, i.e., anchoring pin guide (APG) adapted from the design of the existing denture (**a**) and surgical guide for guided drilling and guided implant placement (**b**). (**c**) Mandibular surgical guide for guided drilling and guided placement of distal implants.

Two maxillary and one mandibular surgical guide were chairside fabricated using a 3D printer (Straumann^®^ CARES^®^ P30+, Institut Straumann AG, Basel, Switzerland) to transfer the virtual plan to the surgical field (Figure 3). The maxillary surgical guide was stabilized by the palatal mucosa and by three bone-anchoring pins (Ø1.3 × 28 mm, positions 11, 15, 23, Fixation Pin, Neodent, Brazil). Drillholes for the pins were prepared using an APG in the form of the conventional denture retro-engineered from CBCT data (Figure 3a). This design supported the correct and careful positioning of the pins and surgical guide (Figure 3b). The mandibular surgical guide was stabilized in position using the residual canines, and used for guided placement of the distal implants, while mesial implants were placed freehand after tooth extraction (Figure 3c).

**Figure 4:**
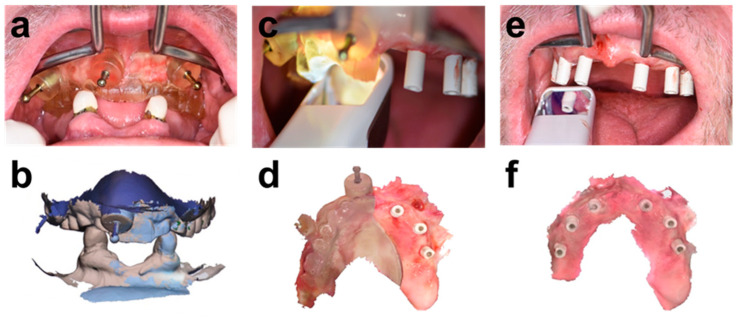
Sequence of performed intra-surgical intra-oral scans (IOSs) for the integrated prosthetic planning. (**a**) APG in place. (**b**) Overlay of the Intraoral Scan 1 (IOS1) of the APG in occlusion with a diagnostic intraoral scan of the conventional denture for positional verification. (**c**) Split-APG and contralateral scan abutments in place. (**d**) Intraoral scan 2 (IOS2) after placement of implants, placement of trimmed APG (“Split-APG”), and contralateral scan abutments. (**e**,**f**) Scanning and resulting IOS 3 of the complete implant base restored with scan abutments.

The correct positioning of the APG (Figure 4a) was validated by overlaying the intraoral scan of the APG in place (IOS1) with the diagnostic initial intraoral scan (Figure 4b). Residual canines were used as a reference for matching these intraoral scans. Likewise, the APG scanned in IOS1 was used as a reference for the relative intraoral position of the diagnostic wax-up for the integrated chairside prosthetic planning workflow.

The flapless guided implant placement was carried out under local infiltration anesthesia (Xylestesin-A with 2% Lignocaine (3M ESPE)) according to the manufacturer’s instructions targeting an insertion torque of ≥35 Ncm. Six implants (BLT, Institut Straumann AG, Basel, Switzerland) were placed in positions 12, 16, 22, and 26 (Ø3.3 × 10 mm), and in positions 14 and 24 (Ø4.1 × 12 mm), respectively, and restored with screw-retained abutments (SRAs) (titanium abutments, Institut Straumann AG, Basel, Switzerland; positions 16, 26: H:1 mm, positions 12, 14, 22, 24: H:2 mm). Healing caps were mounted to bridge any waiting times.

Figure 4 illustrates the sequence of intraoral scans. These scans were integrated into the surgical procedure and used to derive a detailed post-placement prosthetic planning model, including the patient’s soft tissue anatomy and detailed positional information on the implants. These scans included:

1. IOS 1 of the positioned APG (Figure 4a,b)
2. IOS 2 of the patient wearing a bone-anchored “split APG” and contralateral implants restored with SRAs and scan bodies. The split APG was prepared chairside by cutting the APG along the sagittal plane (Figure 4c,d).
3. IOS 3 of the complete implant base restored with scan bodies (Figure 4e,f).

### 2.4. Chairside Immediate Provisionalization

**Figure 5:**
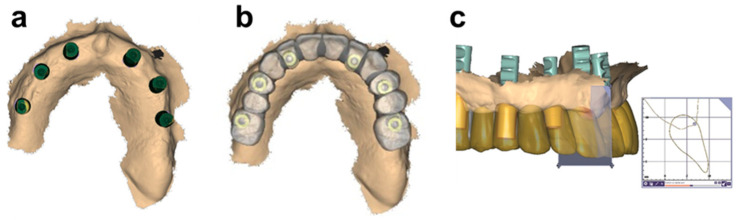
Illustration of the final prosthetic waxing process using a prosthetic model comprising implant positions, soft tissue contours, and soft tissue thickness. (**a**) Soft tissue contour and scan abutments. (**b**) Alignment of provisional prosthetic wax-up on implant base. (**c**) Detailed planning of the cervical prosthetic contours under consideration of the local soft tissue anatomy, i.e., contours and thickness.

The superimposition of these three scans and the diagnostic provisional wax-up allowed for the establishment of an accurate geometric relationship between the maxilla-mandibular relationship, the soft anatomy, the implant-connecting geometry positions, and the provisional diagnostic wax-up. The resulting and refined prosthetic model is illustrated in Figure 5a, and was subsequently used to define the cervical soft tissue interface and connecting geometry of the bridge (Exocad, DentalCAD, exocad GmbH, Darmstadt, Germany). Specifically, as illustrated in Figure 5b, the model was first aligned with the diagnostic wax-up. Subsequently, the prosthetic framework’s emergence profiles and cervical contours were adapted to the local soft tissue anatomy and thicknesses (Figure 5c). Cervical aspects were planned for tight contact without compressing the underlying soft tissue by maintaining a soft tissue thickness of ≥3 mm between the prosthetic contour and the underlying alveolar bone to avoid any soft tissue complications [26].

**Figure 6:**
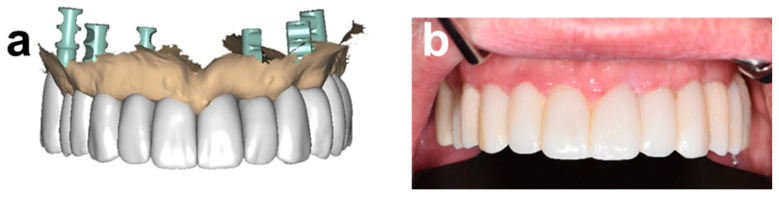
Comparison of the final prosthetic wax-up with the clinical situation 1 week after immediate provisional restoration. (**a**) Final prosthetic wax-up in relation to soft tissue contours. (**b**) Frontal retracted view of immediate provisional.

Figure 6a,b show the final wax-up, and illustrate the adaptation of the soft tissues to the provisional chairside printed prosthesis 1 week post-surgery (CARES^®^ C Series, Institut Straumann AG, Basel, Switzerland).

The mandibular procedure was carried out after the maxillary one, taking place on the same day. In detail, distal implants were placed flapless in positions #34 and #44 (BLT, Institut Straumann AG, Basel, Switzerland, Ø3.3 × 12 mm) using a tooth-retained surgical guide. Subsequently, the residual canines were extracted, and a mucoperiosteal flap was raised in the anterior area. Next, the plane of the alveolar crest was leveled by manual bone reduction, and two anterior straight implants were placed in positions #32 and #42 (BLT, Institut Straumann AG, Basel, Switzerland, Ø3.3 × 10 mm).

An integrated prosthetic workflow was performed similarly to the above described maxillary one. Specifically, scan bodies were mounted on the distal implants after placement and before tooth extraction, and IOS recorded their position. A second IOS was recorded after placement of the anterior implants, flap closure, and after restoring the complete implant base with scan bodies. Registration of these two scans allowed for the establishment of a positional relation between the diagnostic wax-up, the positions of the implant base, and the soft tissue anatomy. The resulting model was used to design the cervical aspects of the chairside printed immediate mandibular restoration.

The provisional and final restoration occlusion was assessed digitally using OccluSense (Dr. Jean Bausch GmbH & Co. KG, Köln, Germany) at 1, 2, and 4 weeks after delivery to identify and eliminate premature contact points. Furthermore, the jaw movement and the temporomandibular joints were clinically examined at the 4 week time point.

## 3. Results

Healing of the soft tissues was uneventful and as predicted. The patient expressed his strong satisfaction with the procedure, and with the aesthetic and functional outcome. He immediately adapted to the temporary and final restorations and self-reported a noticeable improvement in palatal tongue space along with a phonetically-improved ability for pronunciation. The patient further displayed a noticeable change in his behavior and appearance at the one-week reentry, which was potentially associated with an increased level of confidence and self-esteem

**Figure 7:**
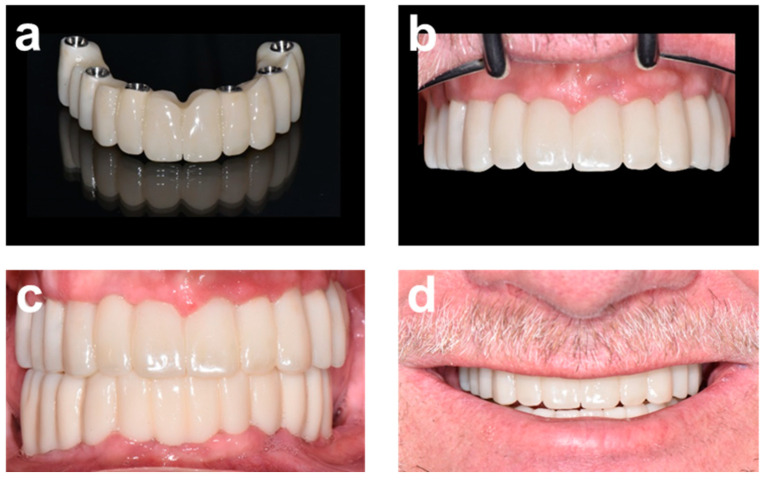
Delivery and aesthetic evaluation of final prosthetic restoration. (**a**,**b**) final full-ceramic bridge before and after delivery. (**c**,**d**) Clinical and aesthetic appearance of final maxillary and mandibular restorations in retracted view and in relation to smile lines, respectively.

The final restoration and clinical outcome are illustrated in Figure 7. A milled multilayer zirconia bridge (IPS e.max ZirCAD Prime, Ivoclar Vivadent AG, Schaan, Liechtenstein) was in-house milled (Ceramill^®^ motion2, Amann Girbach AG, Rankweil, Austria) and delivered six months post-surgery. The fit and occlusion of the provisional prosthesis at reentry were assessed as optimal. Furthermore, the patient expressed his explicit wish to reproduce the design and aesthetic aspects of the provisional restoration for the final bridge. This patient preference corresponds with data from an internal assessment of over 350 patient records that indicate that 97% of treated patients in our clinics wish to directly transfer the aesthetic aspects of the provisional into the final restoration.

The final restoration and clinical outcome are illustrated in Figure 7. A milled multilayer zirconia bridge (IPS e.max ZirCAD Prime, Ivoclar Vivadent AG, Schaan, Lichtenstein) was in-house milled (Ceramill^®^ motion2, Amann Girbach AG, Rankweil, Austria) and delivered six months post-surgery. The fit and occlusion of the provisional prosthesis at reentry were assessed as optimal. Furthermore, the patient expressed his explicit wish to reproduce the design and aesthetic aspects of the provisional restoration for the final bridge. According to internal assessments, this type of choice is in line with the preference of most patients in our clinic, which indicates that 97% of treated patients wish to directly transfer the aesthetic aspects of the provisional into the final restoration with little to no changes.

As evidenced in Figure 7, the provisional and final restoration only displayed minor customization of shade and translucency. Crown morphologies displayed minimal occlusal embrasures and axial curvature. These aspects were defined in conjunction and consent with the patient as part of the DSD. The relatively reduced occlusal embrasures may also assist in reducing any transversal forces to the prosthetic framework and implant base. The patient accepted this profile well, considering that he transferred from a similar low embrasure profile of his conventional dentures [27].

## 4. Discussion

Direct digital immediate IFCD workflows can be considered technically demanding. To reliably deliver functionally and aesthetically successful outcomes, prosthetic and surgical models are required that contain detailed and accurate positional information on the maxilla-mandibular relationship, prosthetic dimensions, the alveolar osseous anatomy, soft tissue architecture, and implant restoration.

**Figure 8:**
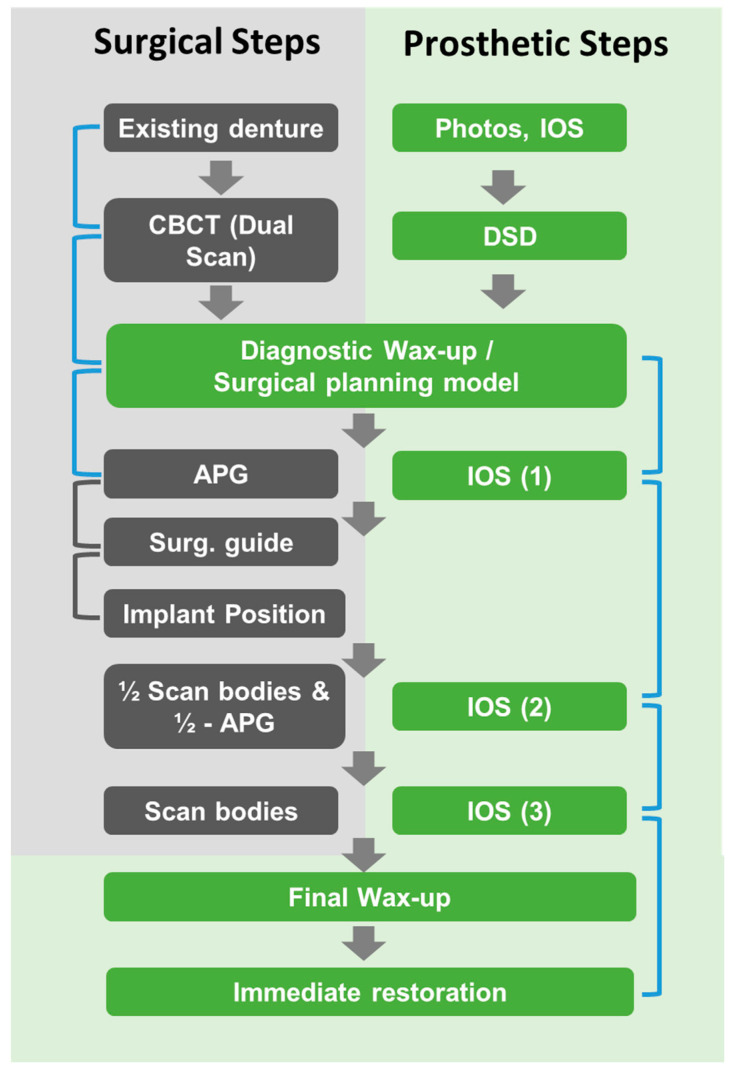
General scheme illustrating the overall treatment concept. Individual steps of the prosthetic procedure are illustrated in green, while surgical steps are illustrated in grey. Individual data sets of the prosthetic and surgical workflows were matched using the positional information of the implants and the original and immediate restorations. Associations of the matched data sets are indicated by blue parenthesis. Positional associations between the APG, surgical guide, and implants are indicated by grey parenthesis. Abbreviations: DSD: digital smile design, CBCT: cone-beam computer tomography, APG: anchoring pin guide, IOS: intraoral scan, Surg. guide: surgical guide.

The presented case report describes the application of an advanced direct digital workflow that can be applied for the immediate full-arch restoration of fully edentulous patients or patients with failing dentition. The approach is schematically summarized in Figure 8 and used an integrated prosthetic workflow that was efficiently incorporated and directly linked to the surgical workflow. This integration was based on transferring the design of the existing conventional denture and associated maxillo-mandibular relationship by replicating it as a reference into the surgical guides. These guides were subsequently used as positional landmarks for the prosthetic models to link and register individual surgical steps created throughout the procedure. As illustrated by the blue brackets in Figure 8, these links between individual data sets indirectly established a geometric and positional relationship between the baseline conventional restoration and the future planned restoration. A further specific characteristic of the workflow and the prosthetic model used was related to the fact that the soft tissue interfacing contours of the planned prosthesis were defined post-placement, and based on the final implant geometry and resulting three-dimensional soft tissue anatomy.

The requirement to combine multiple digital impressions to register the exact implant positions relative to the patient’s alveolar crest anatomy and soft tissue contours may be considered as a common requirement of IFCD digital prosthetic workflows [18,28]. However, the associated registration of individual STL files requires the availability of characteristic landmarks. Many strategies for scan registration have been reported, including the use of temporary mucosa-adhesive fiducial markers, reference bone-anchored fixation pins, temporary maxillary mini-implants, and special custom-made scanning guides [18,20,29]. Compared to these approaches, the registration of individual STL files in the approach described in this paper was achieved by using a split-APG (“½-APG”) or, in the case of the less complex mandibular procedure, residual teeth as landmarks. The APG may be considered of specific importance in the presented workflow as it replicated the existing denture. This design allowed us to directly verify the correct positioning of the APG, i.e., by comparing it to the initial diagnostic intraoral scan of the denture. This intrinsic positional validation may support an improved and accurate implant placement by indirectly ensuring the correct positioning of the surgical guide. The grey brackets in Figure 8 were used to illustrate this relationship. Furthermore, the individual intraoral scans of the APG and split-APG allowed us to relate the position of the final planned restoration and maxillo-mandibular relationship to the situation at the baseline reference. Compared to the literature-reported approaches, the described workflow did not use additional auxiliary materials or custom-made guides that were required to be specifically produced for scan registration. Instead, only existing guides were used, and these were part of the surgical workflow itself.

The strategy to derive the maxillo-mandibular relationship and OVD from an existing removable restoration has been previously described for replicating conventional dentures, as well as for planning implant-based restorative concepts [30,31]. Moura et al. have, for example, directly integrated this strategy into the surgical workflow by using a denture replica-shaped bone-supported drill guide [22]. Michelinakis used a similar prototype-like tissue-supported placement guide that was also used for bite registration as part of the prosthetic workflow [21]. To our knowledge, the presented workflow is the first to use this approach for the soft tissue anatomy-driven chairside design of the immediate provisional as part of an integrated prosthetic workflow.

An accurate transfer of actual implant positions and soft tissue contours to the virtual prosthetic models is imperative for the presented workflow to achieve an adequate final prosthetic fit. Accurate intraoral scans of the entire edentulous arch can be considered a prerequisite to achieving this goal [32]. The soft tissue between fixtures and the absence of characteristic landmarks in these areas render the required alignment of subsequent individual scans especially difficult. The relative mobility of mandibular soft tissues, tongue movement, and the limited space in the distal scanning regions, may designate further potential risk factors for the accuracy of the scans [32,33]. These factors need to be carefully considered during intraoral scanning. During the correct fitting and positioning of the denture, APG and ½ APG represent other crucial aspects that may impact the accuracy of the presented workflow, translating into placement and passive fit inaccuracies. Potential errors can be minimized by verifying the denture’s proper fit, performing necessary relining, and carefully verifying the seating and bite before and during the procedure. One important intrinsic element in the workflow is the option to verify scan and placement accuracy during the matching process of individual intraoral scans. Misalignment of redundant areas between the presented individual intraoral scans may indicate potential scan artifacts and calls for a chairside correction of potential scan errors.

A further important feature of the workflow was the delivery of an immediate provisional pink-free and natural teeth-like white bridge, that was individually adapted to the patient’s facial aesthetics and local soft tissue contours [12]. It must be acknowledged that the defect type of the treated patient was classified as a teeth-only defect. This allowed for such a straightforward restoration. However, recent modern digital smile design concepts and advanced virtual prosthetic planning tools can fully redesign dental arches according to established functional and aesthetic design principles, and validate these designs visually and in real-time [9]. This may also provide some degree of flexibility when extending the concept of white bridges into situations with increased vertical dimensions, traditionally considered as combined defects.

To provide an aesthetically pleasing outcome with an immediate passive fit, pre-surgical diagnostic waxing of the aesthetic and functional contours was combined with, and finalized by, a soft tissue anatomy-driven post-surgical waxing procedure. A combination of digital photographs and intraoral scans was used to guide the DSD and the virtual waxing of the coronal aspects of the prosthesis [10,34]. Coachman et al. recently described this combination as an efficient method for the DSD of a CAD-CAM manufactured immediate restoration [10]. However, the authors required a relatively high amount of pink aesthetics, which supports the advantage of adapting the cervical aspects to the local soft tissue anatomy to deliver a natural tooth-like restoration. In the case described herein, this aspect was developed further by combining this approach with a technique recently introduced by Pozzi et al. [11]. Specifically, the presented methodology allowed us to finalize the cervical aspects of the prosthesis according to the local soft tissue contours and thickness, resulting in a natural teeth-like coronal and cervical design of the delivered bridge.

## 5. Conclusions

The presented case report described the immediate bimaxillary rehabilitation of a patient with failing dentition with a fixed full-arch prosthesis. Guided surgery and an advanced integrated direct digital prosthetic workflow were applied. This workflow may allow for improved prosthetic planning accuracy and clinical efficiency. It also allows us to sequentially adapt the restoration to the patient’s aesthetic and anatomic individual preconditions to result in a natural tooth-like rehabilitation.

## Data Availability

Data sharing does not apply to this case report as no systematic datasets were generated or analyzed during the current study. Any reported underlying data represent patient data, underlie general data protection regulations, and are restricted from sharing.
